# Cytotoxic Effects Induced by Combined Exposure of the Patulin, Ochratoxin A, and Acetamiprid to HK-2 and SK-N-SH Cell Lines

**DOI:** 10.3390/toxins17110563

**Published:** 2025-11-18

**Authors:** Zuoyin Zhu, Hanke Zhao, Xianli Yang, Dongxia Nie, Junhua Yang, Zheng Han

**Affiliations:** Institute for Agro-Food Standards and Testing Technology, Shanghai Academy of Agricultural Sciences, Shanghai 201403, China; zhuzuoyin123@163.com (Z.Z.);

**Keywords:** mycotoxins, pesticides, combined toxicity, dose reduction indices (DRI)

## Abstract

Patulin (PAT), ochratoxin A (OTA), and acetamiprid (ACM) are common food contaminants that frequently co-occur in agricultural products, raising concerns over their cumulative health risks. This study is the first to systematically assess the combined cytotoxic effects of PAT, OTA, and ACM using combination index (CI) and dose reduction index (DRI) models in HK-2 and SK-N-SH cells. All three compounds exhibited dose-dependent toxicity, with potency ranked as PAT > OTA > ACM. In HK-2 cells, PAT+OTA and OTA+ACM showed primarily antagonistic interactions, with synergism observed at low doses. PAT+ACM displayed exposure time-dependent additive effects, while the ternary mixture was mostly antagonistic, with OTA being the dominant contributor. In SK-N-SH cells, most combinations were antagonistic; however, OTA+ACM showed dose-dependent shifts, and the triple mixture transitioned from antagonism to synergism at higher concentrations. OTA and ACM were identified as the main toxicity drivers in all combinations. These findings highlight dose- and cell-specific interactions and underscore the importance of cumulative risk assessment of co-occurring mycotoxins and pesticides in food safety regulation.

## 1. Introduction

Mycotoxins and pesticide residues frequently co-exist as environmental contaminants [1], which could significantly affect food safety, agricultural quality, and human and animal health, and have been positioned as the priority targets in food safety research [2]. Currently, more than 400 distinct mycotoxins have been identified, and these toxins exert a range of deleterious effects, particularly on the kidneys, liver, and intestines [3]. Among different mycotoxins, patulin (PAT) and ochratoxin A (OTA) were detected to be specific infestations of fruits and vegetables, cereals, and other agricultural by-products, consequently inducing compound contamination risks across food chains [4]. A global survey reported a 29% OTA positivity rate in cereals [5], while OTA was found in 53% of grain, fruit, and soy-based products in Canada [6]. PAT contamination was also prevalent, with 57.4% of fruit samples in Pakistan testing positive at levels ranging from 0.04 to 1100 μg/kg [7]. In China, a study of 137 fruit products revealed a 30.7% PAT detection rate, with 7.5% of samples exceeding the maximum residue limits (MRLs) set by the European Union [8]. Acetamiprid (ACM), a commonly used neonicotinoid insecticide, has been detected in multiple matrices, including soil [9], water [10], vegetables and fruits [11], and milk [12]. In Beijing, ACM was detected in 100% of tested fruit and vegetable samples [13], with concentrations in Canadian organic produce ranging from 0.11 to 108 μg/kg [14].

The high prevalence and co-contamination potential of PAT-OTA-ACM in agricultural commodities requires harmonized risk assessment to protect human/livestock health. Recently, there has been increased interest in PAT and OTA’s detection in approximately 75% and 50% of processed cereal food samples and 40% of analyzed samples in Portugal [15], respectively. Co-contamination of mycotoxins and pesticides in Austrian beer lees was found, with 6 to 12 kinds of pesticide residues in each sample [16]. In addition, the monitoring of mycotoxin and pesticide levels showed that co-contamination of OTA and ACM was confirmed in buckwheat samples across production and storage phases in Shanxi Province, China [17]. The pervasive contamination of PAT and OTA in agricultural commodities (fruits/vegetables/cereals), coupled with ACM’s high environmental mobility and matrix penetrability, has positioned combined toxicity assessment as an urgent research priority in food toxicology.

Substantial evidence suggests that PAT causes the nephrotoxicity and hepatotoxicity in human and animals [18]. The mechanism of toxicity involves in the formation of covalent compounds containing sulfhydryl compounds, such as glutathione (GSH) and mercaptoacetate [19]. Glutathione induces the over-production of reactive oxygen species (ROS) in cells, which disrupts their normal physiological function and ultimately leads to apoptosis [20]. The symptoms of OTA poisoning are dose-related, and OTA is the most toxic ochratoxin congener [21], which can lead to renal injury, cancer, and failure [22]. ACM is a highly targeted and potent insecticide that acts as an agonist of nicotinic acetylcholine receptors and effectively controls the endanger of insect pests in agricultural production [23]. Besides of the neurotoxicity, ACM triggers oxidative stress in tissues and damages lipids and proteins through the accumulation of free radicals, which is strongly associated with hepatotoxicity, neurotoxicity, and nephrotoxicity [24,25]. The underlying mechanisms of the toxic effects caused by PAT, OTA, and ACM mainly related to damage of the kidneys. In insects, ACM primarily acts as a neurotoxic substance, which may result in neurotoxic effects such as abnormal release of neurotransmitters and death of nerve cells. However, in humans, apart from the toxic effects on the nervous system, they may induce the nephrotoxicity and hepatotoxicity, and seriously pose a risk to the human health.

Recently, several studies have investigated the combined toxic effects of co-occurring contaminants originating from similar sources [26,27]. Lu et al. [28] demonstrated, using both in vivo and in vitro models, that co-exposure to PAT and chlorpyrifos (CPF) significantly increased overall toxicity compared to individual treatments. In a binary cytotoxicity model, the interaction between OTA and dichlorodiphenyltrichloroethane (DDT) was antagonistic at low concentrations but became synergistic at higher doses [29]. Additionally, co-exposure to OTA and azoxystrobin (AZO) in zebrafish induced more severe toxic effects than either compound alone, as evidenced by elevated oxidative stress, enhanced inflammatory responses, and increased apoptosis-related biomarkers [30]. Despite these findings, comprehensive information on the combined toxic effects and underlying mechanisms of mycotoxin–pesticide co-occurrence remains limited.

In this study, the combined toxic effects of three contaminants, PAT-OTA-ACM, were investigated using human renal tubular epithelial cell line (HK-2) and a human neuroblastoma cell line (SK-N-SH), and these two cell lines are representative of the target organs of these contaminants. The combined toxic effects of PAT, OTA, and ACM were revealed for the first time in five dimensions: duration of action, dose of action, multivariate combinations, combined effects, and dose–contribution ratio to cells. This study aimed to elucidate the interactions between mycotoxins and pesticides impacting the kidney and nervous system, thereby improving comprehension of their cumulative effects on human health and advancing the formulation of appropriate preventive and therapeutic countermeasures.

## 2. Results

### 2.1. Individual Effects of PAT, OTA, and ACM Exposure on Cellular IC_50_ Values

**Figure 1:**
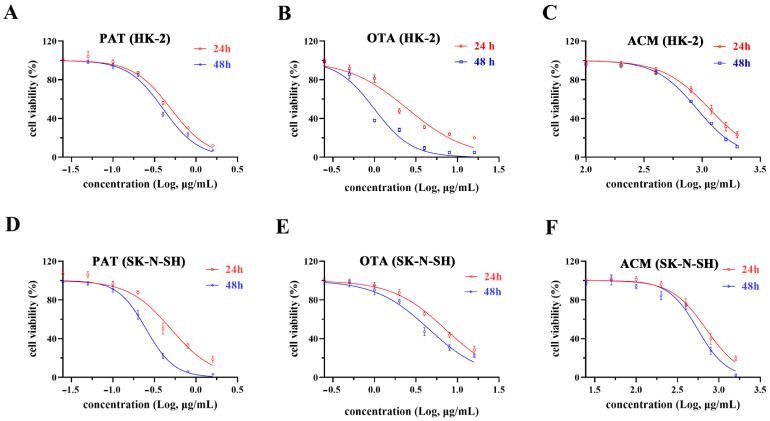
Dose–response curves for the cytotoxicity of PAT, OTA, and ACM in HK-2 and SK-N-SH cells. (**A**–**C**) IC_50_ curves of PAT, OTA, and ACM in HK-2 cells, respectively. (**D**–**F**) IC_50_ curves of PAT, OTA, and ACM in SK-N-SH cells, respectively.

The viability of HK-2 and SK-N-SH cells after PAT, OTA, and ACM treatments was evaluated using the CCK-8 assay kit. Upon exposure to different concentrations of PAT, OTA, and ACM for 24 and 48 h, cell viability decreased in a dose-dependent manner, with differing IC_50_ values for each compound (Figure 1A–F and Table 1). In both cells, the IC_50_ values of the three contaminants after 24 h treatment was significantly higher than that after 48 h, indicating that the longer the duration of action, the lower the IC_50_ value and the greater the toxicity (*p* < 0.05). Based on the IC_50_ values, PAT and OTA were the most highly toxic agents in HK-2 cells, and ACM toxicity was significantly enhanced in SK-N-SH cells. The overall cytotoxicity of the three contaminants in HK-2 and SK-N-SH cells were ranking PAT > OTA > ACM.

### 2.2. Cell Viability of Multi-Mixture of Contaminants

**Figure 2:**
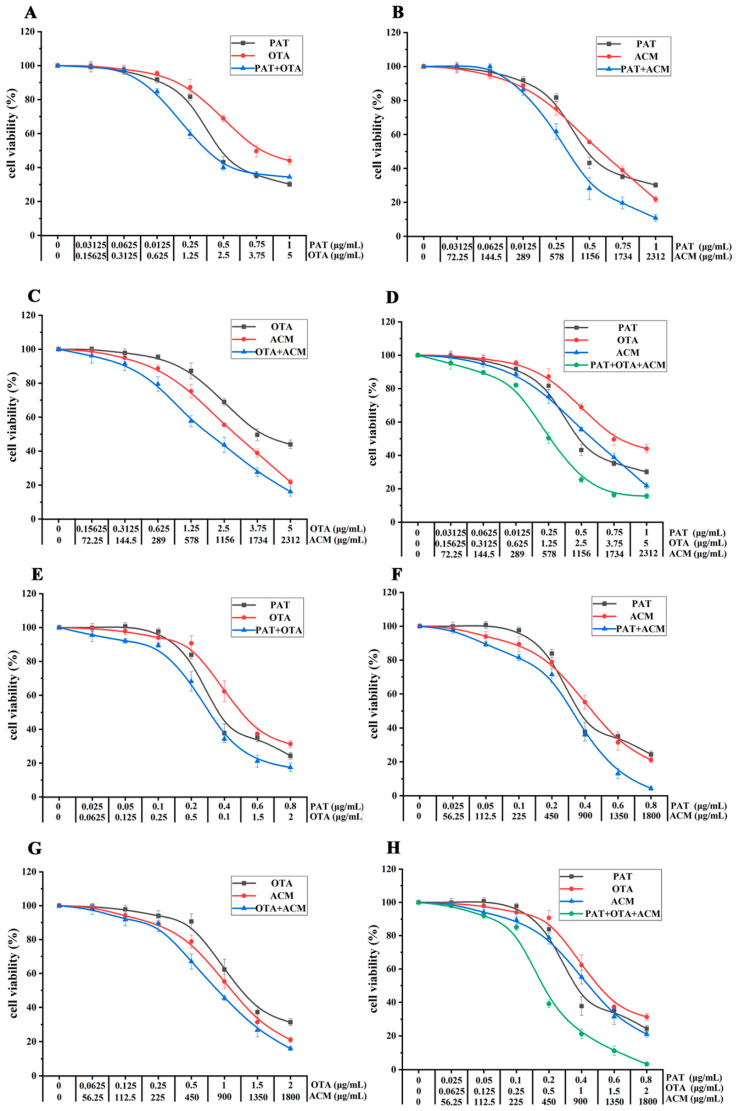
Combined effects of PAT, OTA, and ACM on HK-2 cell viability at 24 and 48 h. (**A**–**D**) show the toxic effects of PAT+OTA, PAT+ACM, OTA+ACM, and PAT+OTA+ACM after 24 h of exposure, respectively. (**E**–**H**) correspond to the same combinations after 48 h of exposure. Cell viability was assessed to evaluate the time- and combination-dependent cytotoxicity of the compounds.

HK-2 cells were treated with four distinct combinations of the three contaminants for 24 h to evaluate the effects of co-exposure on cellular activity. In the PAT+OTA co-exposure group, a more pronounced inhibition of cellular activity was observed at combined concentrations below 1.5 × IC_50_, relative to treatment with PAT or OTA alone. However, at concentrations exceeding 1.5 × IC_50_, the trend in cellular activity followed the order: PAT < PAT+OTA < OTA (Figure 2A). In contrast, co-treatment with PAT and ACM resulted in higher cellular activity compared to the individual treatments when the total toxin concentration was below 0.25 × IC_50_. At concentrations above this threshold, the cellular activity in the co-treatment group was declined than that of the individual treatment groups (Figure 2B). Following OTA+ACM and PAT+OTA+ACM co-exposure groups, the cellular activity was consistently lower in the combination treatments than in the individual exposures, with the dose–response curves for ACM closely resembling those of the combination treatments (Figure 2C,D). Exposure of HK-2 cells to various contaminants combinations for 48 h demonstrated a general increase in cytotoxicity with prolonged exposure, most notably in the ternary combination group. At a combined concentration of 2 × IC_50_, cell viability fell below 5% in both the PAT+ACM and PAT+OTA+ACM treatment groups. Notably, the dose–response profile of PAT in the PAT+OTA group closely aligned with that of combination treatment (Figure 2E). In contrast, the dose–response curves for ACM in the PAT+ACM, OTA+ACM, and PAT+OTA+ACM groups aligned more closely with those of the corresponding combination treatments (Figure 2F–H).

**Figure 3:**
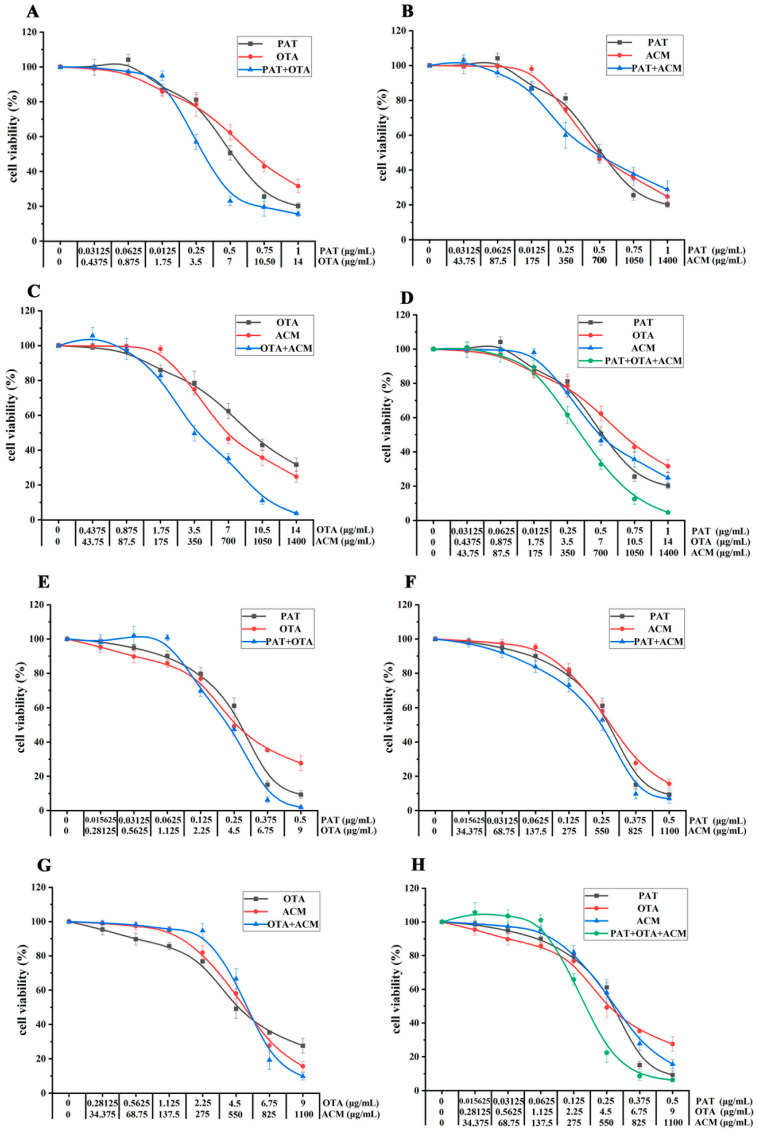
Combined effects of PAT, OTA, and ACM on SK-N-SH cell viability at 24 and 48 h. (**A**–**D**) show the effects of PAT+OTA, PAT+ACM, OTA+ACM, and PAT+OTA+ACM, respectively, after 24 h of exposure. (**E**–**H**) correspond to the same combinations after 48 h of exposure. Cell viability was assessed to explore the time- and combination-dependent cytotoxicity of the compounds.

As shown in Figure 3, the effects of binary and ternary combinations of PAT, OTA, and ACM on the viability of SK-N-SH cells were observed after 24 and 48 h exposures. After 24 h exposure, the co-exposure to PAT and OTA significantly reduced cell viability compared to individual toxin treatments, with the combined concentration exceeding 0.25 × IC_50_ (Figure 3A). Conversely, the PAT+ACM combination elicited a biphasic response, enhancing cell viability at concentrations below 0.0625 × IC_50_ and above IC_50_ (Figure 3B). In OTA+ACM and PAT+OTA+ACM groups, a marked reduction in cell viability was observed above 0.25 × IC_50_ relative to the corresponding single-toxin exposures (Figure 3C,D). Following 48 h exposure, the combinations PAT+OTA and PAT+OTA+ACM resulted in the increase in cell viability below 0.25 × IC_50_ compared to their respective single-toxin counterparts (Figure 3E–H). In contrast, all tested concentrations of the PAT+ACM combination led to the decrease in viability relative to individual toxin exposures (Figure 3F). Notably, the OTA+ACM combination enhanced cell viability below IC_50_, compared to OTA or ACM alone (Figure 3G).

### 2.3. Combined Toxicity Analysis

The differences and similarities in the effects of binary and ternary combinations, as well as variations in exposure duration and dose, were investigated more intuitively and systematically by constructing combination index–effect (CI–Fa) curves.

**Figure 4:**
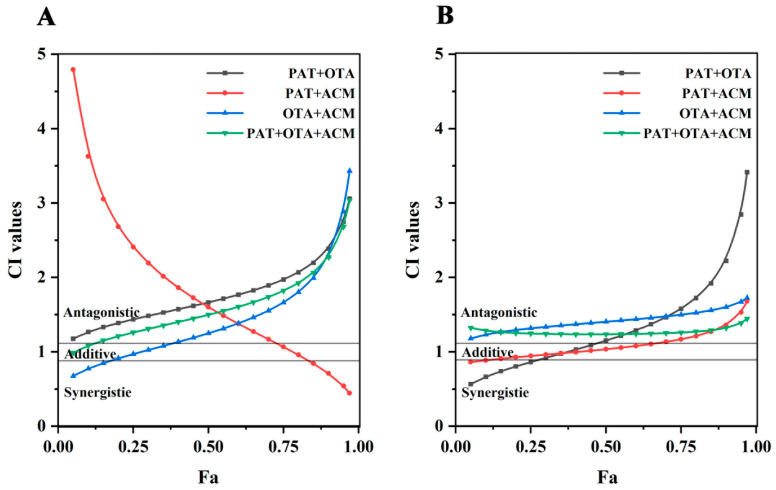
Combined exponential effect curves (CI–Fa) for binary and ternary mixtures of PAT, OTA, and ACM in HK-2 cells. (**A**) Cells were exposed to the compounds for 24 h; (**B**) cells were exposed for 48 h. CI values were plotted against the Fa to evaluate synergistic, additive, or antagonistic interactions. CI values between 0.90 and 1.10 indicated additive effects, CI < 0.90 indicated synergistic effects, and CI > 1.10 indicated antagonistic effects. The horizontal gray solid lines represented the lower and upper boundaries of the additivity zone.

As shown in Figure 4A, the CI–Fa curves for PAT+OTA, OTA+ACM, and PAT+OTA+ACM were the same for 24 h toxicity in HK-2 cells. A dose-dependent increase in antagonism was observed in PAT+OTA group across low, medium, and high doses (1.17 < CI < 3.06). In PAT+ACM group, the change in the combined toxic effect was more pronounced, with a gradual shift from strong antagonism to synergism following low to high doses: antagonism at doses with an induced effect value of Fa < 0.7 (1.16 < CI < 4.80), additivity at doses with 0.75 < Fa < 0.8, and weak synergism at doses with Fa > 0.8. In comparison, the combined toxic effect of the OTA+ACM group was opposite to that of the PAT+ACM group, gradually shifting from synergism to strong antagonism, following the synergism at doses with an induced effect value of Fa ≤ 0.15 (CI < 0.85), additivity at doses with 0.15 < Fa < 0.35, and antagonism at doses with Fa > 0.4 (CI > 1.13). The combined toxicity in the PAT+OTA+ACM group was predominantly antagonistic at doses with Fa < 0.15; the combined effect was shown to be additive (0.9 < CI < 1.08) and antagonistic (CI ≥ 0.15), and the antagonistic effect was more pronounced at higher doses, with the CI value increasing from 1.15 to 3.04.

As shown in Figure 4B, the CI–Fa curves of PAT+ACM, OTA+ACM, and PAT+OTA+ACM were similar after 48 h of multiple co-infiltration of HK-2 cells with PAT, OAT, and ACM. In the PAT+OTA group, there was a gradual shift from synergism to strong antagonism between low to high doses, being synergistic at doses with Fa < 0.3 (CI < 0.9), additive at doses with 0.3 < Fa < 0.45 (0.91 < CI < 1.09), and antagonistic at doses with Fa > 0.5 (1.15 < CI < 3.41). Combined toxicity in the PAT+ACM group was predominantly additive (additive at doses with 0.15 < Fa < 0.75 and synergistic at doses with Fa < 0.15), whereas doses with an induced effect value of Fa ≥ 0.75 and 1.32 ≤ CI < 1.68 were antagonistic. The OTA+ACM group showed antagonistic effects at low, medium, and high doses, 1.18 < CI < 1.73. The PAT+OTA+ACM group showed similar effects to the OTA+ACM group, with weak antagonistic effects at low, medium, and high doses (1.25 < CI < 1.44), respectively. The ternary combination showed weaker antagonistic effects compared to the OTA+ACM group, with a trend of additive or synergistic effects at low doses. Additionally, the antagonistic effect was weaker than that in OTA+ACM group, with an additive or synergistic tendency at low doses. The antagonistic effect was weaker than that in the OTA+ACM group, with an additive or synergistic effects at low doses.

**Figure 5:**
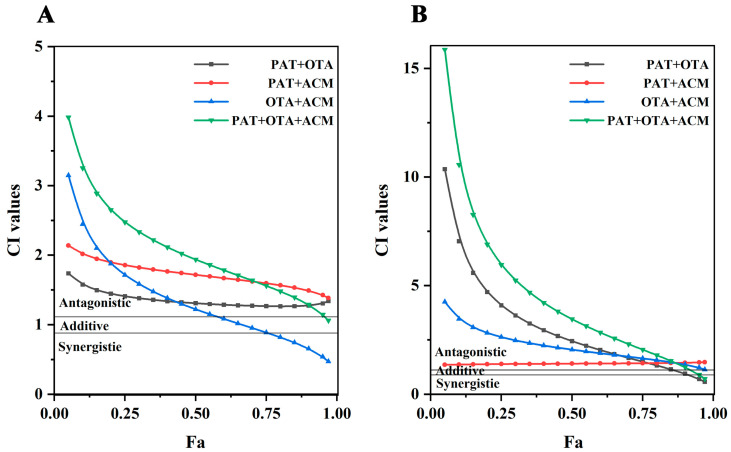
Combined exponential effect curves (CI–Fa) for binary and ternary mixtures of PAT, OTA, and ACM in SK-N-SH cells. (**A**) Cells were exposed to the compounds for 24 h; (**B**) cells were exposed for 48 h. CI values were plotted against the Fa to evaluate synergistic, additive, or antagonistic interactions.

As shown in Figure 5A, after the multivariate combination of PAT, OTA, and ACM treated on SK-N-SH cells for 24 h, the CI–Fa curves of the PAT+OTA and PAT+ACM groups were essentially the same, and then gradually changed from antagonistic to weakly antagonistic from low to high doses, whereas the toxicity of the PAT+OTA group did not change considerably. In contrast, the OTA+ACM group showed a more pronounced change in synergistic toxicity, with a gradual shift from strong antagonism to synergism from low to high doses, with CIs for antagonism ranging from 1.15 to 3.15 at doses with an induced effect value of Fa < 0.55, and additive and synergistic effects at the values of 0.6 < Fa < 0.7 and doses > 0.75. The PAT+OTA+ACM group showed predominantly antagonistic effects at low, medium, and high doses, which gradually diminished from very strong with increasing doses (1.14 < CI < 3.98), with additive effects with Fa = 0.97.

As shown in Figure 5B, the CI–Fa curves of PAT+OTA, OTA+ACM, and PAT+OTA+ACM in SK-N-SH cells were relatively similar after 48 h of the multiple combined effects. From low to high doses, the combined effect of the PAT+OTA group gradually changed from strong antagonism to weak synergy, with CI > 1.1 being antagonistic at doses with an induced effect value of Fa < 0.85, additive at 0.9 < CI < 1.1, and synergistic at greater than 0.9 with a CI < 0.7, for doses between 0.85 and 0.9. The CI–Fa curve of the PAT+ACM group was a straight line close to 1, with CI = 1.35–1.47, mainly showing a weak antagonistic effect. However, the antagonistic effects in the OTA+ACM group gradually became stronger and weaker at low and high doses, respectively. The PAT+OTA+ACM group showed predominantly antagonistic effects at low, medium, and high doses (Fa < 0.9), which gradually diminished from very strong as the dose increased (1.25 < CI < 15.86), and synergistic effects when the effect size was high (Fa > 0.95).

### 2.4. DRI Analyses of Multiple Combined Effects of PAT, OTA, and ACM

**Figure 6:**
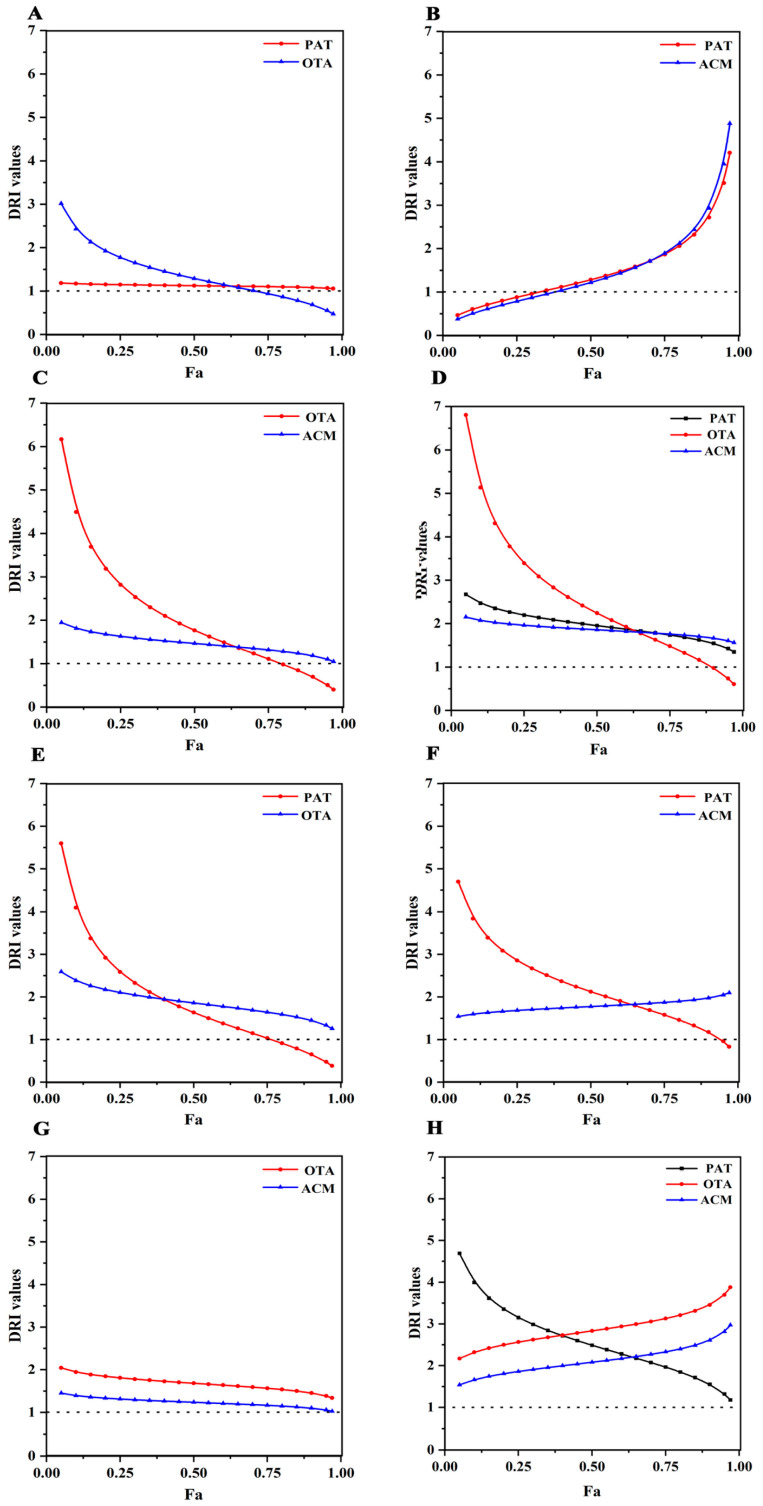
DRI–Fa curves illustrating the combined toxicity of PAT, OTA, and ACM in HK-2 cells. (**A**–**D**) represent the PAT+OTA, PAT+ACM, OTA+ACM, and PAT+OTA+ACM groups, respectively, after 24 h of exposure. (**E**–**H**) corresponds to the same combinations following 48 h of exposure.

DRI values are commonly used to investigate the synergistic effects of combinations of different drugs, and assess the multiplicity of dose reductions that can be maximized by individual drugs when combined [31]. Additionally, it is possible to assess the environmental pollutant co-toxicity, where the DRI corresponds to a multiple of the dose reduction in the individual pollutants when achieving a combined effect. Therefore, we determined the contribution of individual pollutant to the co-toxicity based on the DRI values. Fa–DRI curves were plotted for PAT, OAT, and ACM in binary and ternary combinations based on 24 h isotoxic dose–ratio combinations and CI analysis data (Figure 6). In the PAT+OTA group, the DRI curves of PAT tended to 1 and those of OTA tended to decrease, whereas the changes in OTA on co-toxicity were much greater in HK-2 cells co-induced with PAT+OTA (Figure 6A). In the PAT+ACM group, the trends in DRI for PAT and ACM were essentially analogous, with a greater fold reduction in DRI at higher doses, whereas the effects of PAT and ACM on the PAT+ACM co-combination were comparable (Figure 6B). In the OTA+ACM group, the DRI for ACM did not change considerably, whereas it decreased from 6.12 to 0.60 during the change from low to high OTA doses, indicating that OTA had played a more important role in the combined toxic effects, and dominated the combined toxicity in the OTA+ACM group (Figure 6C). In the PAT+OTA+ACM group, although the DRIs for both PAT and ACM were greater than 1, the change was smaller than that of OTA. In contrast, the dose reduction multiplier for OTA changed more dramatically, with a gradual decrease in the DRI from 6.81 to 0.6 following low to high doses (Figure 6D). Based on the combined exposure of PAT, OTA, and ACM to HK-2 cells for 48 h, the DRIs of PAT and OTA in the PAT+OTA group gradually decreased with the increasing doses. The toxic effect of PAT was more pronounced at low doses, whereas at high doses, the influence of OTA on the combined effect might be greater (Figure 6E). In the PAT+ACM group, the DRI curve of PAT showed a decreasing trend (DRI from 4.7 to 0.84), whereas the DRI of ACM did not change considerably, suggesting that the PAT played a more important role in the PAT+ACM group (Figure 6F). In the OTA+ACM group, the DRI curves of OTA and ACM showed comparable trends. These were not significantly different, with 2 > DRI > 1 suggesting that both pollutants had a comparable influence on the joint effect of this combination (Figure 6G). In the PAT+OTA+ACM group, the DRI curves for PAT and OTA showed a greater magnitude of change, whereas the DRI trend for ACM was smaller, suggesting that PAT and OTA play a major role in the combined effect (Figure 6H). Combining the 24 and 48 h combined toxicity results, it was surmised that the combined toxic effects of the three pollutants on HK-2 cells were mainly due to OTA, PAT, and ACM.

**Figure 7:**
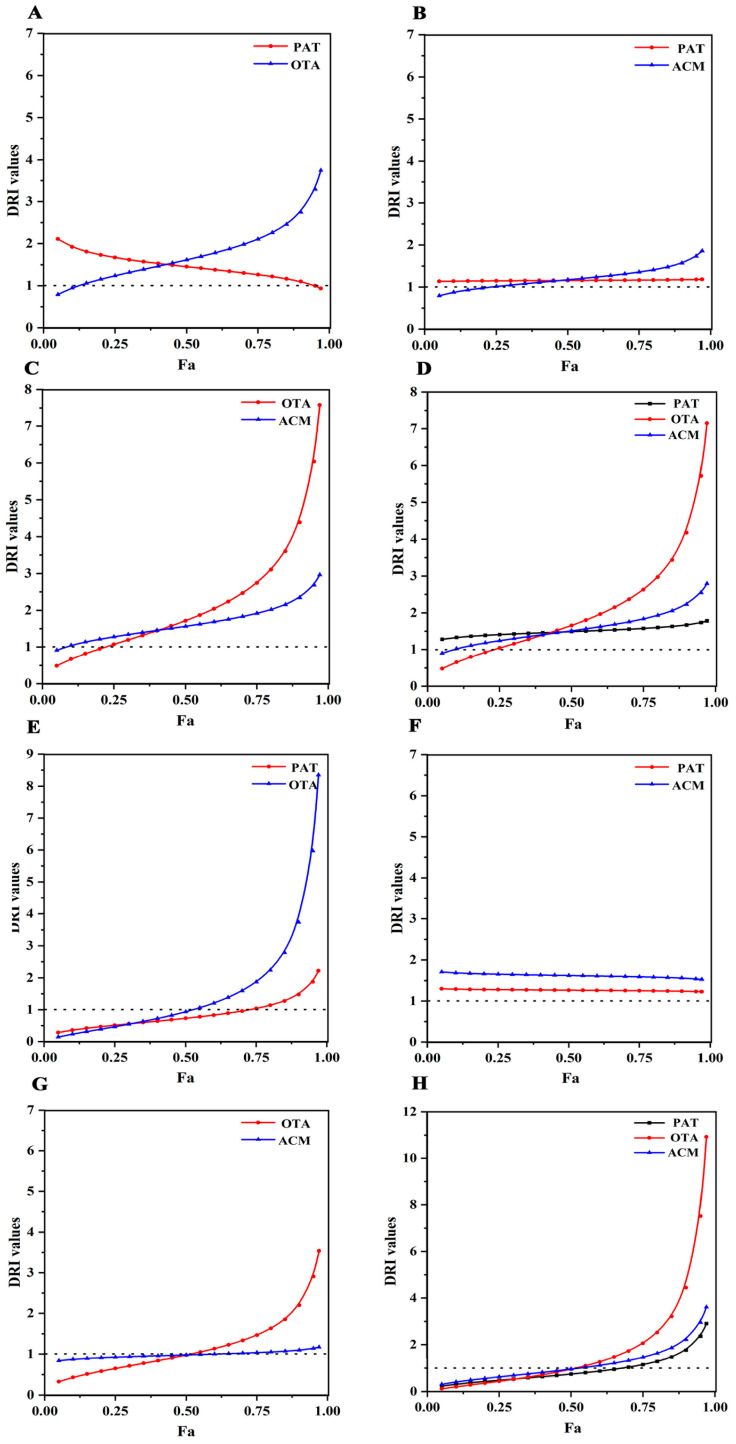
DRI–Fa curves illustrating the combined toxicity of PAT, OTA, and ACM in SK-N-SH cells. (**A**–**D**) represent the PAT+OTA, PAT+ACM, OTA+ACM, and PAT+OTA+ACM groups, respectively, after 24 h of exposure. (**E**–**H**) correspond to the same combinations following 48 h of exposure.

In SK-N-SH cells, Fa–DRI curves were plotted for PAT, OAT, and ACM in binary and ternary combinations based on 24 h induced isotoxic dose–ratio combination data and CI model analysis data. In the PAT+OTA group, PAT and OTA interacted with each other to affect the combined toxicity, and both DRI were greater than 1. The DRI curves continued to decrease after PAT and increased after OTA (Figure 7A). In the PAT+ACM group, the DRI curve for PAT remained essentially unchanged, whereas the DRI curve for ACM continued to increase, and the effect of ACM on the combined effect was greater in the PAT+ACM combined toxicity (Figure 7B). In the OTA+ACM group, the DRI curves for OTA and ACM continued to rise, whereas the DRI increased from 0.49 to 7.58 along with the change from low to high OTA doses (Figure 7C). In the PAT+OTA+ACM group, the DRI curves for PAT presented the less change, while the DRI curves for OTA and ACM showed a larger trend. In contrast, the dose reduction multiplier for OTA was more dramatic, with a gradual decrease in DRI from 0.48 to 7.15 along with low to high doses, followed by ACM (Figure 7D).

After exposure to SK-N-SH cells with a multivariate combination of PAT, OTA, and ACM for 48 h, the DRI values of PAT and OTA in the PAT+OTA group gradually increased with increasing doses; the DRI value of OTA was larger than that of PAT, whereas the change in the OTA-exposed group was greater in the PAT+OTA group (Figure 7E). In the PAT+ACM group, the DRI curves of PAT and ACM did not change considerably; however, the DRI value of ACM was always larger than that of PAT, indicating that the effect of ACM played a more important role in the PAT+ACM group (Figure 7F). In the OTA+ACM group, the DRI curve for ACM remained essentially unchanged, whereas the DRI curve for OTA continued to rise. The DRI value for OTA was greater than 1 when Fa > 0.5, suggesting that OTA had a greater impact on the joint effect after the 50% toxic effect in the OTA+ACM group (Figure 7G). In the PAT+OTA+ACM group, the DRI curves of PAT, OTA, and ACM changed less before the 50% effect, whereas the DRI curves of OTA and ACM trended higher than those of PAT at the 50% effect, suggesting that OTA and ACM played a major role in the combined effect (Figure 7H). Combined with the 24 and 48 h composite toxicity results, we assumed that ACM and OTA, followed by PAT, played a significant role in the SK-N-SH cellular composite toxicity of these three contaminants.

## 3. Discussion

PAT, OTA, and ACM, the representative mycotoxins and pesticide residues frequently co-detected in foods, could cause the damage to human kidneys and nervous system, and affect the normal physiologic functions [32]. In this study, HK-2 and SK-N-SH cell lines were employed to systematically assess the combined cytotoxic effects of PAT, OTA, and ACM, and to quantify the individual contribution of each compound to the observed mixture toxicity. Cell viability assays and IC_50_ values demonstrated that PAT, OTA, and ACM, either individually or in combination, induced a dose-dependent reduction in cell viability across both cell models. The rank order of cytotoxic potency was consistent between different cell lines, following the order: PAT > OTA > ACM. This ranking was consistent with previous studies indicating that PAT exhibited the highest cytotoxicity, following by OTA and ACM [33,34,35], in which, IC_50_ values of PAT and OTA in Caco-2 cells were 15.95 μmol/L and 145.36 μmol/L, respectively. In addition, the 24 h IC_50_ value of ACM in SK-N-SH cells was approximately 10.6 mmol/L [36,37]. These findings suggest that the three contaminants may interact in a dose- and time-dependent manner, displaying either synergistic or antagonistic effects. This highlights the need for further mechanistic investigations to elucidate their combined toxicological actions. Previous studies have shown that co-exposure to mycotoxins and pesticides can simultaneously trigger key toxicological pathways, including oxidative stress, mitochondrial dysfunction, and apoptosis, which may underlie the dose-dependent cellular toxicity observed in our study [38].

Up to now, studies investigating the combined toxicity of mycotoxins and pesticides remained limited, with most studies focusing on the co-exposure to the compounds such as CPF, DDT, AFs, and ZEN [39]. In the present study, the cumulative toxicity of PAT, OTA, and ACM was elicited for the first time. The co-exposure of PAT and OTA predominantly exhibited antagonistic interactions in HK-2 cells, although a synergistic effect was also observed at lower doses. This finding was consistent with previous reports that the co-exposure to OTA and OTA at low dosage induced the synergistic cytotoxicity in porcine renal cell lines (LLC-PK1) [40]. Interestingly, in Caco-2 cells, the PAT+OTA combination exhibited a dose-dependent interaction, with synergism evident at low concentrations and antagonism at higher doses, and OTA played a more critical role in mediating the combined toxicity [36]. The PAT+ACM group mainly showed an additive effect, and the exposure time played a more important part in the combined effect. Similarly, the exposure to DON and OTA, in particular, was toxic to MA-10 Leydig cells, which was enhanced by co-exposure to pesticides [29]. The OTA+ACM group mainly showed antagonism, and it is worth noting that it showed a synergistic effect at a low dose for 24 h. The combined effects of PAT+OTA+ACM and OTA+ACM were similar, and the main manifestation was antagonism. Similarly to the antagonism toxicity induced by the combination of OTA and ACM, a recent study on the combined toxicity of AFB1 and CPF demonstrated that the co-contamination caused an antagonistic cytotoxic effect in HepG2 cells [41]. Furthermore, DRI-based analyses of binary and ternary mixtures in HK-2 cells revealed that all three contaminants contributed substantially to the overall toxicity, with PAT and ACM playing secondary yet significant roles.

In SK-N-SH cells, PAT+OTA mainly showed antagonism, and a synergistic effect was observed at a high dose for 48 h treatment. Our results showed that the OTA + ACM group mainly exhibited antagonistic effects, whereas a high-dose synergistic response emerged after 24 h of treatment. This synergism may be attributable to an additive intensification of toxicity, as both OTA and pesticides have been reported to induce mitochondrial dysfunction and oxidative stress-mediated injury in neuronal cells or the nervous system during individual exposures, accompanied by apoptosis [42,43]. Another study found that PAT showed higher toxicity than citrinin (CIT) on SH-SY5Y cells, irrespective of the incubation time [44]. In the binary combination of OTA and DDT, it was observed with antagonism at low concentrations and synergism at high concentrations [29]. Furthermore, one limited study has demonstrated that combined exposure to OTA and azoxystrobin (AZO) was more toxic to zebrafish than these compounds alone [30]. The PAT+OTA+ACM group was antagonistic at low doses, while it was synergistic at high doses. Following the increase in the toxic effect, it gradually changed from antagonism to synergism. Similar studies have indicated that DON+Cd was presented in combined toxicity studies at different times, treated in HT-29 cells; combined toxicity ranged from antagonism to synergism, with increased cytotoxicity, activated MAPK/AP-1, and increased ROS levels [45]. The DRI curves showed that ACM and OTA played a major role in the combined toxicity of the three pollutants in SK-N-SH cells. Collectively, these findings underscored the critical need to investigate co-exposure effects of mycotoxins and pesticides across diverse biological models and exposure scenarios, which would be essential for accurately characterizing human health risks associated with complex contaminant mixtures in environment.

## 4. Conclusions

This study is the first to systematically investigate the combined cytotoxic effects of PAT, OTA, and ACM on HK-2 and SK-N-SH cells. All compounds exhibited dose-dependent toxicity, with the potency ranking as PAT > OTA > ACM in both cell lines. In HK-2 cells, PAT+OTA, OTA+ACM, and the triple mixture primarily showed antagonistic effects, while PAT+ACM exhibited exposure time-dependent additivity. OTA was the dominant contributor to overall toxicity, followed by PAT and ACM. In SK-N-SH cells, most combinations were antagonistic, although OTA+ACM showed dose-dependent variability, and the triple mixture shifted from antagonism to synergy at higher concentrations. OTA and ACM were identified as the main toxicity drivers in all combinations. These findings reveal cell-specific and dose-dependent interactions, emphasizing the need for joint risk assessment of co-occurring mycotoxins and pesticides, and providing insights to inform monitoring and regulatory strategies in agricultural and environmental settings.

## 5. Materials and Methods

### 5.1. Chemicals

The cell lines of HK-2 and SK-N-SH cells were kindly provided by Procell Life Science and Technology Co., Ltd. (Shanghai, China). Cell Counting Kit-8 (CCK-8) was supplied by Shanghai BioScience Co., Ltd. (Shanghai, China). Minimum Essential Medium (MEM), Dulbecco’s Modified Eagle’s Medium-F12 (DMEM/F-12), fetal bovine serum (FBS), phosphate-buffered saline (PBS), 0.25% Trypsin-EDTA solution and Dimethyl sulfoxide (DMSO) were purchased from Beijing Solarbio Science and Technology Co., Ltd. (Beijing, China).

The standards of PAT, OTA, and ACM were purchased from Pribolab (Qingdao, China) with the purity higher than 99.00%. Stock solutions of contaminations were prepared in DMSO and kept at −20 °C. As established, the final concentration of DMSO in the medium was ≤0.1% (*v*/*v*). All other chemicals, unless specified, were purchased from Sigma-Aldrich Pte. Ltd. (Sigma, St. Louis, MO, USA).

### 5.2. Cell Culture

The HK-2 and SK-N-SH cells were cultured in MEM and DMEM/F-12, respectively, each supplemented with 10% FBS, 100 U/mL penicillin, and 100 U/mL streptomycin. Cells were maintained in 25 cm^2^ culture flasks under a humidified atmosphere containing 5% carbon dioxide (CO_2_) at 37 °C.

### 5.3. Individual and Combined Cytotoxicity Assessment

The cytotoxicity of individual contaminations was evaluated using dose–response curves, from which half-maximal inhibitory concentration (IC_50_) values were calculated. Contaminant concentration and exposure duration were identified as the primary determinants influencing cytotoxic responses. Concentrations employed in the binary and ternary combination assays were selected based on IC_50_ values obtained from individual treatments in HK-2 and SK-N-SH cell lines.

Due to the high binding affinity of OTA for serum proteins, all treatments were conducted in medium supplemented with 2% FBS to minimize potential interference with toxicological outcomes [46,47]. For cytotoxicity evaluation, 100 μL of cell suspension (8 × 10^3^ cells/well) was seeded into 96-well plates. Upon reaching approximately 80% confluence, the culture medium was replaced with fresh medium containing varying concentrations of PAT, OTA, ACM, or their combinations. Cells were then incubated for 24 or 48 h, after which cell viability was assessed. Concentrations used for individual mycotoxin exposures are provided in Table S1, while those for binary and ternary mixture exposures are listed in Table S2.

### 5.4. Cell Viability Assessment

Cells were diluted to a density of 8 × 10^3^ cells/well and seeded in 96-well culture plates. After 24 h incubation, all cells were exposed to various concentrations of individual or combined contaminants and allowed to grow in an incubator for 24 h and 48 h, respectively. The cytotoxicity of the two different cell lines was determined using a CCK-8 kit according to the manufacturer’s instructions. After treatment, 10 μL CCK-8 solution was added to each well (10% culture volume) and incubated for 2 h at 37 °C. Absorbance values (*A*) were measured at 450 nm using a microplate reader (Thermo Fisher Scientific, Waltham, MA, USA). All treatments were performed in six replicates, and cell activity was calculated as follows:

$$Cell\;Viability(\%)=\frac{A\left(experiment\;\right)-A(blank)}{A\left(control\right)-A(blank)}\times \;100$$

### 5.5. Combined Index Analysis of Contamination Mixtures

The dose–effect relationship for PAT, OTA, and ACM was calculated using the neutralization equation proposed by Chou and Talalay [48].

$$\frac{fa}{fu}={\left(\frac{D}{Dm}\right)}^{m}$$

*D* was the concentration of the contamination, *fa* was the cell proliferation inhibition rate, and *fu* was the unaffected fraction (*fu* = 1 − *fa*). *Dm* was the median effect dose for the 50% inhibition of cell proliferation. *m* was the coefficient indicating the shape of the dose–effect relationship (*m* = 1, *m* > 1, and *m* < 1 each indicated hyperbolic, sigmoidal, and flat sigmoidal dose–effect curves). The method employed the *Dm* parameter to measure the cytotoxicity of individual contamination.

When the composition of contaminations extended to binary or ternary, the interactions of multiple contaminations were assayed by means of combination index model (CI) based on the median effect principle, as the CI values calculated according to a generic formula:

$${n_{\left(CI\right)}}_{x}=\sum_{j=1}^{n}\frac{{\left(D\right)}_{j}}{{\left(D_{x}\right)}_{j}}$$

^*n*^(*CI*)_*x*_ referred to *n* kind of contaminant combined cell inhibition rate for x% of composite index, (*D*)_*j*_ was referred to the joint action of *n* type of toxin to a total dose of x% cell inhibition rate, and (*D*_*x*_)_*j*_ referred to the combination of each contamination effect itself when dose of x% cell inhibition rate. Overall, the types of combined effects represented by CI = 0.9–1.1, CI < 0.9, and CI > 1.1 were additive, synergistic, and antagonistic, respectively. The specific classification criteria and corresponding symbols are shown in Table 2.

In addition, the impact of contaminants on the combined effects was assessed using the dose reduction index (DRI). DRI indicated a multiplicative reduction in the dose required to produce the same effect when the contaminant was mixed compared to when used alone. DRI > 1 indicated that the dose reduction was favorable to the combined effect, and DRI < 1 indicated that the dose reduction effect was unfavorable [49]. The DRI was calculated as follows:

$$n_{{\left(CI\right)}_{x}}=\sum_{j=1}^{n}\frac{{\left(D\right)}_{j}}{{\left(D_{m}\right)}_{j}}=\sum_{j=1}^{n}\frac{1}{{\left(DRI\right)}_{j}}\;\;or\;{\;(DRI)}_{j}=\frac{{\left(D\right)}_{j}}{{(D}_{m}{)}_{j}}\;$$

### 5.6. Statistical Analysis

The IC_50_ values of the three different contaminations exposed to the two cell lines was calculated by GraphPad Prism 9.4.0 software. All data were assayed by ANOVA using IBM SPSS Statistics 20 software, LSD significance test, *p* < 0.05 for significant difference, *p* < 0.01 for highly significant difference, and all the results data were expressed as mean ± SEM. Origin 2021 was used for performing the line plotting. ComboSyn 3.0.1 software (Inc., Paramus, NJ, USA) was used for calculating the indexes of the CI and DRI about three contaminants.

## Figures and Tables

**Table 1:** IC_50_ Values in HK-2 and SK-N-SH cells exposed to PAT, OTA, and ACM.

Cells	Times (h)	PAT (μg/mL)	OTA (μg/mL)	ACM (μg/mL)
HK-2	24	0.5 ± 0.005 ^a^	2.5 ± 0.049 ^c^	1156.3 ± 38.70 ^a^
	48	0.4 ± 0.006 ^b^	1.0 ± 0.021 ^d^	900.2 ± 4.1 ^b^
SK-N-SH	24	0.5 ± 0.024 ^a^	7.0 ± 0.357 ^a^	699.8 ± 34.41 ^c^
	48	0.25 ± 0.001 ^c^	4.5 ± 0.415 ^b^	549.5 ± 31.58 ^d^

Data are presented as the means ± SEM, *n* = 6. ^a^, ^b^, ^c^, and ^d^: in each column, values without a common letter differ significantly (*p* < 0.05).

**Table 2:** Descriptions and symbols of the degrees of combined toxicity grading.

CI Value	Description	Graded Symbols
<0.10	Very strong synergism	+ + + + +
0.10–0.30	Strong synergism	+ + + +
0.30–0.70	Common synergism	+ + +
0.70–0.85	Moderate synergism	+ +
0.85–0.90	Slight synergism	+
0.90–1.10	Nearly additive	±
1.10–1.20	Slight antagonism	–
1.20–1.45	Moderate antagonism	– –
1.45–3.30	Common antagonism	– – –
3.30–10.0	Strong antagonism	– – – –
>10.0	Very strong antagonism	– – – – –

## Data Availability

The original contributions presented in this study are included in the article/Supplementary Materials. Further inquiries can be directed to the corresponding authors.

## Supplementary Materials

The following supporting information can be downloaded at: https://www.mdpi.com/article/10.3390/toxins17110563/s1, Table S1. Concentrations of the three individual contaminants used for 24- and 48 h exposures. Table S2. Concentrations of contaminants used in binary and ternary exposure experiments for 24 h and 48 h treatment durations.
