# Supplementary material for: Cytotoxic Effects Induced by Combined Exposure of the Patulin, Ochratoxin A, and Acetamiprid to HK-2 and SK-N-SH Cell Lines
Source: Toxins (Basel). 2025 Nov 18;17(11):563. doi: 10.3390/toxins17110563 (PMC12656219; doi:10.3390/toxins17110563)
Supplement: Supplementary file 1 [file toxins-17-00563-s001.zip › toxins-3986470-supplementary.pdf]

Cytotoxic Effects Induced by Combined Exposure of the Patulin, Ochratoxin A, and Acetamiprid to HK-2 and SK-N-SH Cell Lines

Zuoyin Zhu, Hanke Zhao, Xianli Yang, Dongxia Nie, Junhua Yang * and Zheng Han *

Institute for Agro-Food Standards and Testing Technology, Shanghai Academy of Agricultural Sciences, Shanghai 201403, China; zhuzuoyin123@163.com (Z.Z.)
* Correspondence: yangjunhua@saas.sh.cn (J.Y.); hanzheng@saas.sh.cn (Z.H.)

Table S1. Concentrations of the three individual contaminants used for 24- and 48 h exposures.

Cells Lines	PAT (μg/mL)	OTA (μg/mL)	ACM (μg/mL)
HK-2	0.025	0.25	100
	0.05	0.5	200
	0.1	1	400
	0.2	2	800
	0.4	4	1200
	0.8	8	1600
	1.6	16	2000
SK-N-SH	0.025	0.25	25
	0.05	0.5	50
	0.1	1	100
	0.2	2	200
	0.4	4	400
	0.8	8	800
	1.6	16	1600

Table S2. Concentrations of contaminants used in binary and ternary exposure experiments for 24 h and 48 h treatment durations.

Cells Lines.	24 h			48 h		
	PAT (μg/mL)	OTA (μg/mL)	ACM (μg/mL)	PAT (μg/mL)	OTA (μg/mL)	ACM (μg/mL)
HK-2	0.03125	0.15625	72.25	0.025	0.0625	56.25
	0.0625	0.3125	144.5	0.05	0.125	112.5
	0.125	0.625	289	0.1	0.25	225
	0.25	1.25	578	0.2	0.5	450
	0.5	2.5	1156	0.4	1	900
	0.75	3.75	1734	0.6	1.5	1350
	1	5	2312	0.8	2	1800
SK-N-SH	0.03125	0.4375	43.75	0.015625	0.28125	34.375
	0.0625	0.875	87.5	0.03125	0.5625	68.75
	0.125	1.75	175	0.0625	1.125	137.5
	0.25	3.5	350	0.125	2.25	275
	0.5	7	700	0.25	4.5	550
	0.75	10.5	1050	0.375	6.75	825
	1	14	1400	0.5	9	1100

To investigate the cytotoxic effects of binary and ternary combinations of PAT, OTA, and ACM, fixed-ratio mixtures were prepared based on the principle of isotoxicity, utilizing the individual IC_{50} values of each mycotoxin following 24 h and 48 h exposures in HK-2 and SK-N-SH cell lines. In HK-2 cells, the concentration ratios (PAT: OTA: ACM) for the 24 h and 48 h mixtures were 1: 5: 2312 and 1: 2.5: 2250, respectively. In SK-N-SH cells, the

corresponding ratios were 1: 14: 1400 and 1: 18: 2200. Comprehensive details regarding the concentration gradients and specific compositions of each treatment subgroup are provided in Table S2.
